# The Prevalence and Outlook of Doping in Electronic Sports (Esports): An Original Study and Review of the Overlooked Medical Challenges

**DOI:** 10.7759/cureus.48490

**Published:** 2023-11-08

**Authors:** Stanisław Słyk, Marcin Zarzycki, Kacper Grudzień, Gabriel Majewski, Michał Jasny, Izabela Domitrz

**Affiliations:** 1 Department of Medicine, Medical University of Warsaw, Warsaw, POL; 2 Department of Neurology, Medical University of Warsaw, Warsaw, POL; 3 Department of Neurology, Bielanski Hospital, Warsaw, POL; 4 Department of Psychiatry, Bielanski Hospital, Warsaw, POL; 5 Faculty of Medicine, Jagiellonian University Medical College, Kraków, POL; 6 Clinic of Pediatric Neurosurgery, University Children's Hospital, Kraków, POL; 7 Department of Humanities and Social Sciences, Józef Piłsudski University of Physical Education in Warsaw, Warsaw, POL

**Keywords:** well-being, public health, motivations, esports, doping

## Abstract

Background

The first electronic sports (esports) tournament was recorded in 1972, and since then, gaming leagues and tournaments with prizes have been established. Nowadays, the commercialization of competitive gaming may drive players to cheat their way to success and neglect their physical and mental well-being. The issue is all the more vital, as it is often overlooked by classically educated doctors, including sports medicine specialists. The aim of this study was to investigate the current situation of doping in esports and the future of anti-doping actions in this field, as well as to present a more generalised approach and to point out and discuss other possible health risks associated with the rising popularity of esports.

Methods

A standardised online survey was published in a social media group for Polish fans and people associated with esports. Two hundred and forty-one responses were collected and subjected to a statistical analysis. Only filled-out forms containing answers to all of the questionnaire's questions from people considering themselves regular players of either League of Legends (LoL) and/or Counter-Strike: Global Offensive (CS:GO) were considered viable. The study group was divided into amateur and professional players. The calculation of test power was done post hoc to determine whether the data collected were of sufficient quality to be used further. The normal distribution was assessed using the Shapiro-Wilk test. Then, between-group differences comparing the data results from the questionnaire were analysed with Mann-Whitney U tests and Chi-square tests. The significance level was set at p <0.05. Then, the literature was screened for relevant articles.

Results

The majority of gamers (85.5%), despite most of them being amateurs, strive to achieve the best results when playing. Borderline legal substances are commonly used, including energy drinks (97.8%), coffee (81.6%), beer (29.7%), herbs (15.7%), and available medicines (15.1%), while more than half the participants have heard about situations during tournaments involving the use of substances forbidden by the World Anti-Doping Agency (WADA). In most answers, there were no significant differences between professionals and amateurs. Statistically significant differences were observed in the following areas: the usage of legal stimulants in the responders' environments; outlooks on implementing more strict anti-doping regulations; and the perceived usage of forbidden doping substances in a tournament setting.

Conclusions

E-competitors suffer from a plethora of both physical and mental health problems. These issues may be more prevalent than generally thought and include repetitive strain injuries, sleep disorders, anxiety, and depression. The largest esports organisations have started to implement measures to provide a holistic approach to esports healthcare; however, it remains a distant dream for most amateurs and aspiring e-athletes.

## Introduction

Electronic sport, or Esport, has been around since 1972 when 24 students competed in the first recorded esports tournament. It was not until the late 1990s, though, that the first gaming leagues and tournaments with substantial prize pools were established [1]. Nowadays, esports is bigger than ever. The International 2018 had a total prize pool of over $25 million [2]. The League of Legends (LoL) 2018 World Championship had a peak viewership of over 205 million, with an average of over 46 million [3]. Huge sponsorship deals and franchising provide fertile ground for further growth. All of the above-mentioned factors contribute to the immense pressure esportsmen might experience both in the strictly competitive tournament setting and during training sessions, which in turn drives them to neglect their physical and mental well-being. The issue is all the more vital, as it is often overlooked by classically educated doctors, including sports medicine specialists. This issue, in tandem with the lack of standardised healthcare catering to professional gamers and the lack of routine checkups or real preventative measures introduced, can rapidly evolve into a global eruption of esports-related issues. According to data, out of 4500 respondents to an online survey who played at least once a week, carried out in multiple countries, as many as 36% would quit their jobs if they could provide themselves with a sustainable income from playing video games, and more than 57% of male gamers aged between 18 and 35 years would like to become professional players [4]. As the numbers show, this sector of the entertainment industry is fast-growing, and we might experience a future explosion of candidates for professional e-competitors, necessitating the need to expand our knowledge on the possible health problems that pertain to this demographic.

The growing popularity and profits from esports also result in more and more reasons for players to cheat their way to success. There have been reports of numerous such instances. In 2014, there was a huge match-fixing scandal, which resulted in seven individuals getting banned from events sponsored by Valve [5]. There have been many instances of using hacks in Counter-Strike: Global Offensive (CS: GO), the most recent of which involved an Indian player being indefinitely banned [6]. Esports is also not free from doping. Although not much is known about its scale, reports have emerged from the community. For instance, in 2015, a high-level CS: GO player revealed that many people used Adderall during tournaments [7].

The Cambridge English Dictionary defines doping as “the act of giving a person or animal drugs in order to make them perform better or worse in a competition” [8]. The earliest records of doping date from 668 BC, when athletes studied the effects of special diets on their performance [9]. The ancient Greeks were also known to use alcohol as part of their training routine and experiment with stimulants developed from various plants and fungi. With rapid advances in science and medicine in the 19th and early 20th centuries, new means of doping were developed, including the use of hormones like testosterone. Since 2004, doping has been strictly regulated, as the World Anti-Doping Agency (WADA) annually publishes the List of Prohibited Substances and Methods [10]. It was also in 2004 that WADA first established the World Anti-Doping Code, which summarises anti-doping policies around the world and harmonises them, with its most recent iteration being published in 2021 [11]. This code proves the standard dictionary definition to be lacking and outdated. According to WADA, doping is the occurrence of anti-doping rule violations, which include not only the use or attempted use of prohibited substances but also, inter alia, the presence of such substances or their markers in athletes’ samples, evading or tampering with the control, or even possession and trafficking of the substances. The battle with doping is a dynamic process, as new attempts to bypass regulations are always being detected. There are also new disciplines of competition in which regulators have to discover how to best tackle the issue of doping.

Esports is becoming extremely popular and is even being considered as a possible Olympic discipline. There are also numerous betting sites that encourage users to bet on esports matches, opening up a whole new realm of risks. Thus, it is crucial to deepen and declutter our knowledge. The aim of this study was twofold. On one hand, we conducted a questionnaire survey with the intention of investigating the current situation of doping in esports. As this is just a drop in the ocean of possible new health-related problems associated with the rapidly growing esports industry, we would also like to present a more generalised approach and point out and discuss other possible health risks associated with the rising popularity of esports among both e-athletes and the wider population, possibly laying the groundwork for future studies in this field.

## Materials and methods

In order to answer research questions, an online survey was created. Two of the most popular (national and worldwide) competitive games (as of 2019) were chosen to be investigated: LoL and CS:GO. The cross-sectional study was designed, and the distribution of the survey was conducted via social media. It was published in a Facebook group created for Polish esports fans, gathering more than 23,000 players. The data were collected over a period of one week, from November 15 to November 22, 2018. The survey was reposted once, on November 20, 2018, in order to increase the response rate. The questions covered four general areas: demographic information with the type of revenue source, game and time spent on gaming, usage and beliefs about listed substances, opinions on doping, and related topics. The idea behind creating the questionnaire was to make a simple question-and-answer design sufficient to provide a general public perception of the researched issue.

The study was conducted at the Medical University of Warsaw, Warsaw, Poland. At the time the study was conducted (2018), Bioethics Committee approval was not required in Poland for surveys in which participants could take part voluntarily via the Internet. Personal data have been anonymized and do not allow for the identification of the subjects. Participation in the survey was voluntary, and its purpose was made clear before the questions were disclosed. We therefore assumed that each participant agreed to the processing of the obtained data in this study.

The study group was divided into amateur and professional players based on self-declaration, as there are no universally accepted objective distinctions between these two groups of gamers. The inclusion criteria for the study were to regularly play one or both of the previously mentioned video games. Responders who did not answer all questions properly (by checking options and/or properly filling the response gap) were excluded. Also, answers that contained irrelevant answers (like inappropriate vocabulary or off-topic responses) were ignored. There were no other exclusion criteria.

Responses were collected using the online survey application Google Forms (Google LLC, Mountainview, CA) and then entered into Microsoft Office Excel 365 (Microsoft Corp., Redmond, WA) for further investigation. The statistical analysis was conducted with Statistica 13.1 (Quest Software Inc., Aliso Viejo, CA). The statistically significant differences in answers were examined with variables based on the questions:

1. Gender

2. Do you practice esports professionally?

3. Are you a member of an esports organization?

4. Have you ever participated in esports tournaments?

5. Is competition in esports a source of your income?

The G*Power 3.1.9.7 program (Heinrich-Heine-Universität Düsseldorf, Düsseldorf, Germany) was used to calculate the power of the tests used. The calculation was done post hoc to determine whether the data collected were of sufficient quality to be used further. The minimal acceptable power of the test (1-β error probability) was set at 0.8.

The normal distribution was assessed using the Shapiro-Wilk test. Then, between-group differences comparing the data results from the questionnaire were analyzed with Mann-Whitney U tests and Chi-squared. The significance level was set at p <0.05.

Then, the literature was screened for relevant articles. As there are few studies on the issue, a traditional systematic review and meta-analysis were not possible. Medical databases were screened and then supplemented with general and industry-specific media reports.

## Results

Two hundred and forty-one answers were collected. The mean age of all participants was 19.8 years (median=18), for amateurs, 19.8 years (median=18.5), and for professionals, 19.8 years (median=18). Female responders were statistically significantly older (women's median age=21.1 years vs. men's median age=18.9 years; p<0.05). The mean number of hours a week spent playing CS:GO/LoL was 21.6 hours, with a standard deviation of 15.8. The full results of the survey are presented in Table 1.

**Table 1:** Online survey questions and results

Question	Answer	N (%)
Demographics		
Game	Counter-Strike: Global Offensive (CS:GO)	113 (46.9%)
	League of Legends (LoL)	128 (53.1%)
Sex	Male	221 (91.7%)
	Female	20 (8.3%)
	Other	0 (0%)
Do you practice esports professionally?	Yes	35 (14.5%)
	No	206 (85.5%)
Are you a member of an esports organization?	Yes	38 (15.8%)
	No	203 (84.2%)
Have you ever participated in esports tournaments?	Yes	167 (69.3%)
	No	74 (30.7%)
Is competition in esports a source of your income?	Yes	34 (14.1%)
	No	207 (85.9%)
What is your main motivation for playing CS:GO/LoL?	Fun	180 (74.7%)
	Recognition/popularity	29 (12%)
	Achieving best results	147 (61%)
	Money	26 (10.8%)
	To get away from it all	85 (35.3%)
	Job	1 (0.4%)
Usage and attitude towards doping		
Do you think that there is a doping problem in esports?	Yes	61 (25.3%)
	No	86 (35.7%)
	Hard to say	94 (39%)
Do people from your environment use such legal substances?	Yes, many people	114 (47.3%)
	Yes, few people	59 (24.5%)
	No	50 (20.7%)
	Hard to say	18 (7.5%)
If so, which ones?	Coffee	151 (81.6%)
	Energy drinks	181 (97.8%)
	Beer	55 (29.7%)
	Herbs	29 (15.7%)
	Available medicine	28 (15.1%)
Do you think that the use of some of these substances should be forbidden?	Yes	52 (21.6%)
	No	141 (58.5%)
	Hard to say	48 (19.9%)
Do you think that the use of such substances should be controlled in any way?	Yes	70 (29%)
	No	106 (44%)
	Hard to say	65 (27%)
Have you ever heard about a situation during tournaments involving the use of substances considered by The World Anti-Doping Agency as forbidden, or illegal ones (cocaine, amphetamine, Adderall, etc.)?	Yes	133 (55.2%)
	No	108 (44.8%)
In your gaming environment, have there been instances of using forbidden substances during tournaments?	Yes	26 (10.8%)
	No	215 (89.2%)
Have you heard about any actions (e.g. of tournament organizers) aimed at counteracting possible doping attempts?	Yes	90 (37.3%)
	No	151 (62.7%)
Have you ever heard about people punished for doping in esports?	Yes	76 (31.5%)
	No	165 (68.5%)
Do you think that there should be more restrictive anti-doping actions in esports?	Yes	107 (44.4%)
	No	68 (28.2%)
	Hard to say	66 (27.4%)
Could medical examination by sports physicians during tournaments help increase the fairness of games in esports?	Yes	146 (60.6%)
	No	48 (19.9%)
	Hard to say	47 (19.5%)
Could anti-doping control, such as taking blood and urine samples, help increase the fairness of games in esports?	Yes	140 (58.1%)
	No	56 (23.2%)
	Hard to say	45 (18.7%)
Which of these motivations most frequently push players to use doping in esports?	Pursuit of victory at all costs	204 (84.6%)
	Lack of anti-doping control	68 (28.2%)
	Low effectiveness or imperfection of anti-doping controls	29 (12%)
	Negligence of tournament organizers concerning anti-doping control	56 (23.2%)
	Connivance in the environment – doping is not considered reprehensible and is generally accepted	54 (22.4%)
	Lack of punishment for people involved	53 (22%)
	Money	4 (1.7%)
	Stupidity	1 (0.4%)

Statistically significant differences, with the power of the test close to one, concerning the current situation of doping in esports and the future of anti-doping control in esports were revealed in only three questions:

1. Do people from your environment use such legal substances?: More members of esports organizations chose positive answers (86.8%) than amateurs (69.0%), p<0.05.

2. Do you think that there should be more restrictive anti-doping actions in esports?: Fewer members of esports organizations chose positive answers (26.3%) than amateurs (47.8%), p<0.05.

3. In your gaming environment, have there been instances of using forbidden substances during tournaments?: More players earning with the help of esports chose positive answers (20.6%) than amateurs (9.2%), p<0.05.

The full data for the aforementioned questions is presented below, in Table 2.

**Table 2:** Full data regarding the statistically significant questions

	Do people from your environment use such legal substances?			Do you think that there should be more restrictive anti-doping actions in esports?			In your gaming environment, have there been instances of using forbidden substances during tournaments?	
Do you practice esports professionally?	No	Hard to say	Yes	No	Hard to say	Yes	No	Yes
No	46	17	143	55	56	95	190	16
Yes	4	1	30	13	10	12	25	10

No other questions presented statistically significant differences between the amateurs' and professionals' answers.

## Discussion

Overview

With the rapid growth of esports, the issue of doping may present an immediate threat to the health of both professional e-athletes and amateurs. About 35.7% of the respondents do not think that there is already a problem, while 25.3% are positive about it. The large percentage (39%) of “hard to say” answers suggests that there is significant uncertainty and a field for debate about the role of doping in esports. It can challenge researchers to attempt to gain the confidence of the participants in such discussions [12] and for them to answer honestly. In comparison with traditionally defined sports, athletes also have insufficient knowledge about doping [10] to be able to respond.

About 74.7% of the respondents play for fun. Even though many of them are amateurs, over 60% log in to achieve the best results. Similar results have been gathered by Seo in 2016 [13], who identified improving one’s skillfulness and becoming better as one of the key elements of esports ethos. This is in line with our later finding that the largest reason pushing people over the line of doping use is the pursuit of victory at all costs. This mechanism can also be observed in traditional sports. As a study carried out in 2005 by Özdemir et al. has shown, some of the major reasons behind doping use are rising to the task: matching the criteria for team recruitment and enhancing one’s overall performance [14]. This issue has also been pointed out by Morente-Sánchez and Zabala [15].

On the other hand, popularity is a reason for 12% of respondents to play but not cheat (no results). Similarly, even though 10.8% of the participants said that money is the reason they play and 14.1% treat esports as the source of their income, only four people (1.7%) pointed out money as the reason to use doping. This stands in contrast to Morente-Sánchez and Zabala’s findings [15], who pointed out that financial gain and recognition were good enough reasons to use doping for 74% and 24% of participants, respectively. These findings may be the result of the immaturity of the esports scene. As mentioned before, it has only recently entered a stage with large prize pools and sponsorship deals, and it is still not certain what it will look like in the future. This lack of stability makes it impossible to predict if there is going to be more money involved, and therefore it is a very risky motivation to cheat.

Popularity in esports is often achieved through different means than just achievements and performance. Out of all the Entertainment and Sports Programming Network (ESPN) power rankings for the LoL 2018 World Championship [16-20] and the top 20 players list [21], only one makes it to the top five most popular players by Twitter followers (as of August 2018) [22], and none achieve top viewership on Twitch outside of the official Riot Games and respective regional league channels. Therefore, doping is not needed for players to achieve large popularity, as factors other than performance seem to be important, such as the feeling of affecting live streams, the suspense that derives from expecting something unexpected to happen in live streams, and the sharing of dramatic developments that occur in live streams [23].

There are many ways in which athletes try to increase their performance. A short case study was published on a popular cycling website [24], and it proved that even though there are legal substances that improve short-term (coffee) or long-term (creatine) performance, the side effects outweighed the gains. However, in a discipline as unique as esports, similar stimulants could possibly impact game results. Avoiding being distracted and staying focused were listed as two of the main attributes of successful e-athletes [25]. The participants of our questionnaire acknowledged this fact, with 50.2% agreeing that this may indeed be the case and 71.8% admitting to knowing about people in their environment who use substances of this kind.

Unsurprisingly, the most popular stimulants among gamers are coffee and energy drinks. Caffeine has been proven to increase perception and alertness [26], helping with focusing on the task at hand. It also offers a delay in fatigue and an increase in endurance [27], crucial for esports matches, which sometimes last for many hours (the single longest LoL competitive game lasted for almost 95 minutes [28], while players sometimes have to play five-game series). Even remaining focused during esports training might prove challenging, as each year e-athletes train (on average) 5.28 hours per day at the elite level [29]. Caffeine also appeared on the list of substances banned from the Olympics numerous times [30]. Even though caffeine in the form of coffee seems to be less effective than doping with pure caffeine [27], its effects cannot be overlooked, especially with players drinking other caffeinated beverages, like energy drinks. Thus, even though caffeine is currently not considered illegal for athletes, some regulations might be reasonable for esports.

Similarly, beer has been pointed out as possible legal doping. Its sedative effect [31] may help to ease the nerves or help in some games requiring a high level of calmness. Players also mention herbs and widely available medicine as possible ways of legal doping. This heterogeneous group of substances is very hard to analyze, as various medications might have very different effects on the body. However, this should be looked into more closely to find out which drugs are most popular and if they should be regulated or forbidden. A great example of why this is necessary has already been mentioned, with many players using prescription drugs like Adderall during high-level tournaments.

These legal substances might have a significant effect on players’ performance, and it should be discussed whether some of them should be banned or regulated. Even though traditional sports are currently not forbidden, esports is so unique that it might be reasonable to at least put it under discussion. This is illustrated by the disagreement among our participants about whether these substances should be banned or controlled.

The current situation of doping in esports is not limited to borderline legal substances. More than half of our participants have heard about situations during tournaments involving the use of substances forbidden by WADA. Worryingly, 10.8% have also experienced the use of such substances during tournaments in their close gaming environment. This suggests that, even though some players and organizers may be reluctant to admit it, the issue is present and not just limited to news and rumours. On the other hand, over one in three of the respondents confirms that there have been actions aimed at counteracting doping attempts, while 31.5% have heard about instances of people getting punished for using forbidden substances.

Our participants tend to agree, with almost half of them thinking this way, that more restrictive anti-doping actions should be implemented, while about 60% agree that medical examination, maybe even taking blood or urine samples, could help increase fairness in esports. Over one in five of them also agree that it might be due to a lack, or low effectiveness, of current anti-doping controls or a lack of punishment that makes players decide to cheat. There does not seem to be an organized effort against doping in esports, as most teams are self-regulating. However, with growing support from the National Anti-Doping Agency (NADA) [32], such actions may be necessary, and a universal, clear set of rules might be implemented in the future.

The price of striving to perform

Aside from the reasons to use doping in tournaments themselves, e-athletes are also subjected to prolonged training sessions, as mentioned above. As a consequence, they may be prompted to use performance-stimulating substances to be able to focus for extended periods of time. Although direct studies on the prevalence of this phenomenon among esportsmen are lacking, such conclusions can perhaps be extrapolated from statistics on energy drink consumption among gamers as a whole. For example, one article, published by Meehan in 2020, evidences regular consumption of caffeine-containing drinks in 26% of polled gamers in the Asia-Pacific region [33].

Among the chief consequences of exposure to straining training regimens, e-competitors suffer from a plethora of both physical and mental health problems.

Starting with physical disorders, these include mainly mechanical injuries to the musculoskeletal and peripheral nervous systems. Some examples include head and neck pain [34], lower back pain syndrome [35], upper limb disorders (including hands) [36,37], and even lower extremity injuries [38], although these pertain mostly to more casual-oriented video games that require physical movement (e.g., Wii Sports, 2006) [38]. In this case, musculoskeletal disorders may also entail nerve entrapment syndromes, such as carpal tunnel syndrome, tennis elbow, and Guyon canal syndrome [39]. These disorders are secondary to cases of tendonitis in the carpal tunnel, Guyon canal syndromes, and lateral epicondylitis in tennis elbow [39].

As rare as these may seem, these injuries may be more prevalent than is generally thought. One study pointed towards the alarming prevalence of gaming-associated physical ailments, such as wrist and back pain (respectively 36% and 41%) among collegiate e-sportsmen, yet only 2% of the surveyed population who have experienced any medical symptoms sought medical care [35].

As e-athletes produce up to 400 fine motor movements to stabilize and move the wrist, elbow, and shoulder joints, these structures and adjacent tissues are susceptible to repetitive strain injury (RSI). This type of damage is driven by inflicting repeated, frequent motions (causing friction) on soft tissues; these, in turn, promote inflammation and pain symptoms in the affected anatomical area [39].

As far as esports players’ mental well-being is concerned, firstly, it has to be established that most professional esports organizations have a gaming house. It is a place where players coexist for certain periods of time in order to train together and improve their synergy. In most cases, these are houses in which players live together for extended periods of time [40]. This led us to the assumption that e-athletes may be subjected to elevated levels of stress, similar to the case of remote work from home (WFH). One of the studies shows that workers who had to work from home during COVID-19 were more likely to have increased stress levels because of social isolation. Moreover, family-work conflicts (or, in this case, teammate-training conflicts) decrease WFH productivity overall [41]. Spending so much time with the same people every day may cause burnout and worsen one’s well-being. There are no dedicated studies on this matter for e-athletes, but men who work offshore in the oil industry and spend all day in a unigender environment, similar to professional players in a gaming house, show increased levels of anxiety and overall worse general mental health results in the General Health Questionnaire (GHQ) scores. E-athletes spend all their time in gaming houses and don’t have the time to maintain relationships with their families, which may influence their mental state and cause mental illnesses like anxiety or depression. Similar mechanisms have been evidenced in Mexican migrant workers, who feel disconnected from their families, which in turn elevates the level of anxiety and may cause depression [42].

Secondly, the sleeping schedule of players is also disturbed. In one study, it was established that the median total sleep time for e-athletes from the United States, South Korea, and Australia was under seven hours per day. Although the median sleep latency was under the clinical cut-off of 30 min, approximately half of the participants exceeded the clinical cut-off for insomnia and had excessive daytime sleepiness. The median sleep onset was about 3:43 a.m., and the wake time was about 11:24 a.m. [43]. In another study, Korean e-sport athletes reported significantly delayed sleep phases compared to the control group (a difference of 2.10 hours, p < 0.001). The professional players’ group reported substantially lower sleep quality and scores for feeling refreshed upon awakening, higher depression scores, and a significantly higher proportion of individuals with clinical symptoms of depression compared to the general population (p <0.01) [44]. The e-athletes also have a clinical depression cut-off score of ≥16. The Center for Epidemiologic Studies Depression Scale (CES-D) score correlated well with the number of awakenings, wake after sleep onset (WASO), and daily training time [43].

Strenuous training times are no less problematic; according to one study, Korean players train longer than US participants by 7.3 hours and by 8.6 hours than Australian participants, totalling 13.4 hours per day. The Koreans train, on average, from 1:07 p.m. to 4:45 a.m. In comparison, the Australian teams train, on average, from 5:30 p.m. to 10:15 p.m., whereas the US teams generally start their training earlier, starting at 11:48 a.m. and finishing at 6:12 p.m. The same study found that increased training time may increase the risk of depression [43]. Additionally, potentially weekend oversleeping is associated with higher odds of mood, behavioural and mental disorders, suicidality, tobacco smoking, and poor perceived mental and physical health, with odds ratios (OR) ranging from 1.34 to 2.01 [45]. Moreover, frequent gamers report more problems with sleeping (with weekly relapses) than light gamers or non-gamers (once a month) [46]. The same study pointed out that soft drinks are more likely to be consumed by heavy gamers than other groups [46]. Among young adults, there is a substantial tendency to drink more harmful drinks, e.g., alcohol. About 60% of them admitted to consuming more than five standard portions monthly. The differences, when compared to light gamers and non-gamers, were statistically significant [46].

It also has to be stressed that e-athletes are under psychological pressure similar to that of traditional professional athletes. The authors showed that esports players faced 51 different stress factors, including communication problems and concerns with competing in front of live audiences. It resembles the mental challenges experienced by pro athletes, including footballers and rugby stars, in high-profile tournaments. The main stressor was identified as intra-team criticism, which causes anxiety for some players because they may potentially be dropped from the team. The same issue occurs in classic sports [47]. Most e-athletes' coping strategies are focused on avoidance strategies during competitive play, and there was a lack of effective problem- and emotion-focused strategies used during gameplay [47]. This strategy had a negative influence on performance and life comfort among adolescent swimmers during the competitive season [48]. The lack of proper support may cause an increased rate (approximately three to five times larger than the general population) of depressive symptoms, higher levels of perfectionistic concern, and social phobia, such as that shown in young Danish and Swedish elite football players [49]. Proper cognitive behavioural therapy and cognitive restructuring could help decrease perfectionism and improve performance. It could also prevent the development of performance decrements as well as depressive symptoms [49].

Finally, these issues are further exacerbated by other social factors. It has been shown that Korean e-athletes live in a hierarchical environment, especially in the workplace [50]. The data gathered in 2012 showed that 75.8% of Korean workers admitted to having been psychologically bullied (e.g., abusive language, excessive invasion of privacy). They also reported: verbal abuse (51.6%), semi-forced participation in dining events (33.0%), discrimination on the grounds of educational background, appearance, etc. (24.2%), bullying (21.8%), ignored contributions (15.8%), and sexual harassment (15.1%). Furthermore, 84.6% of workers indicated that they “tolerated the situation”. The most common reasons given by the participants included: “I thought that it was part of the organization's culture (61.4%)” and “I thought I could endure it” (27.0%) [51].

Similar problems appear in esports teams. For instance, Seo Jin-hyeok (known as Kanavi), a 19-year-old LoL player who became known as a suspected victim of ‘coercive transfer to China', signed a contract with his team, Griffin, that contained many law-breaking clauses. These terms included, but were not limited to: “If you're sick and cannot make it for more than 30 days, then we will be eligible to terminate your contract immediately.” "If 1. is applied, then you will not be allowed to find another team for another year.” "If we believe your current form is disappointing, then we will be allowed to terminate your contract at any time." "If communication cannot be reached with the player, then the player will be immediately kicked out of the team, along with a $42,000 fine and all of your remaining wages given back to the team.” "After the end of your contract, all the intellectual property rights and trademark rights related to the Griffin brand that were put in during your time with the team related to you must all be paid." [52]. This supports the hypothesis that e-athletes may be subjected to similar or even more strenuous situations than the rest of society. In addition, esports organizations, especially in the East, have numerous teams, which include the main team, academy, and possibly a few more lower-level divisions for the up-and-coming players [53]. Thus, especially the younger competitors, they may experience immense pressure to play well and climb the organizational ladder. In turn, main-team e-athletes may experience fear of being replaced with someone younger after just a few weak performances. Thus, their mental health may be endangered. One study about the influence of workplace stress on mental health proves that subjective stress resulting from “too much competition” may cause mental health problems (GHQ score ≥17) approximately two to four times more often [54].

All of the above emphasizes the need to provide e-athletes with a structured form of prevention-oriented medical care, along with clearance for participation in tournaments and routine checkups. Sadly, it is not the standard currently, and most professional players are relegated to seeking medical help on their own [35], should the need arise. Some top-tier teams, though, have employed medical professionals as permanent staff members in order to provide their competitors with tools to maintain their well-being and hopefully prolong their eligibility for tournament participation.

The largest esports organizations take care of their e-athletes by employing professional psychologists to teach players how to deal with confidence issues, inadequate coping strategies, harassment, difficulty separating gaming and life, and a lack of self- and team development [55]. Moreover, these organizations take care of the e-athletes’ daily routines to maintain their mental and physical health. They hire chefs and nutritionists to prepare food as per the dietary needs of each individual. Some gaming houses even have gyms for physical training, and some of the best players have their personal trainers [56].

Such practice was put into effect in teams FC Schalke 04 and Excel Gaming, who, under sports psychologist Fabian Broich, instigated a basic framework for workplace hygiene for players, including healthy dietary schedules, practice hours limitations, physical exercise, and chat breaks. Although objective analyses of the effectiveness of this approach are lacking, the competitors subjected to it report being content with these methods, going as far as naming their contemporary environment as "the most healthy [esports] team in Europe right now”, in the words of Marc Robert “Caedrel” Lamont [57]. This, however, remains a distant dream for most aspiring e-athletes, who are still being gated from the holistic approach to satisfactory sports healthcare by their insufficient status, barring them from facilities provided by elite teams.

Limitations

The questionnaire was addressed to a subgroup of players who not only play either game regularly but also participate in the discourse regarding them online, possibly skewing the results of our study by highlighting responses from only the most dedicated players.

The survey was published in a group for Polish fans of the games, which may not be representative of the international intersubjective consensus regarding our subject of investigation.

We have managed to gather valid responses from only 1% of members of the Facebook group, which most likely was due to the voluntary, internet-based nature of this survey. In order to be able to fill out this form correctly, one needed to take their time to click the link leading to the questionnaire and dedicate some of their free time to reading its contents, which may have acted as a deterrent for some potential surveyees.

Moreover, we didn’t succeed in achieving parity between male and female respondents, as were the demographics of the players of these games in general and the members of the esports-oriented Facebook group.

## Conclusions

This study emphasises the importance of recognising the key differences and similarities between esports and other traditional disciplines. Understanding the unique environment that comprises the shell of practising for and participating in online tournaments can still be a daunting challenge for the general public, be it an esports fan or a general practitioner, coming into contact with e-athletes, competitors, and amateurs requiring medical attention.

With large groups within society expressing interest in taking up gaming professionally, it is vital to create a safe and sustainable environment for them to thrive. The above-mentioned issues should find their reflections in the appropriate future legislation seeking to ensure just that.
